# Risk Factors for Mild Cognitive Impairment (MCI)

**DOI:** 10.3390/brainsci7090117

**Published:** 2017-09-11

**Authors:** Andrea Mary Loftus

**Affiliations:** School of Psychology and Speech Pathology, Curtin University, Bentley 6102, Western Australia, Australia; andrea.loftus@curtin.edu.au; Tel.: +61-8-9266-5120

It was has been my pleasure to have acted as the guest editor for the Brain Sciences Special Issue on Mild Cognitive Impairment (MCI). The unprecedented worldwide size of the ageing population and the associated increased prevalence of dementias has resulted in an explosion of clinical, neuropathological, and biomarker research. Much of the recent research has been directed towards identifying predictive markers of dementia; and the presence, nature, and severity of MCI are emerging predictors of progression to dementia.

MCI is increasingly acknowledged as a prodromal feature of many of the dementias, including Alzheimer’s, Lewy-Body, and (more recently) Parkinson’s. Distinguishing between MCI and the cognitive impact of normal, healthy aging is a challenge. The concept of ‘non-normative’ cognition is often applied to gain a sense of whether an individual is experiencing cognitive decline, but variability in the number and nature of cognitive tests administered, sources of normative data, and cut-off criteria (1SD, 2SDs, etc.) compound the mystery surrounding MCI for many clinicians and researchers. In light of its potential to predict progression to dementia, the accurate and early identification of MCI is of great importance.

The articles in this special edition address a range of issues in relation to the diagnosis, classification, and impact of MCI, and offer a variety of experiences by which MCI may be further understood. The breadth of articles in this special edition offers an insight into researcher and clinician understandings and interpretations of MCI, and enhances the knowledge and experience of this domain.

